# The Role of Kt/V and Creatinine Clearance on Assisting Optimization of Serum Phosphorus Levels among Patients on Peritoneal Dialysis

**DOI:** 10.34067/KID.0000000618

**Published:** 2024-10-11

**Authors:** Jaime Uribarri, Murilo Guedes, Maria Ines Diaz Bessone, Lili Chan, Andres de la Torre, Ariella Mermelstein, Guillermo Garcia-Garcia, Jochen Raimann, Thyago Moraes, Vincent Peters, Constantijn Konings, Douglas Farrell, Shuchita Sharma, Adrian Guinsburg, Peter Kotanko

**Affiliations:** 1 Renal Division, Department of Medicine, Icahn School of Medicine at Mount Sinai, New York, New York; 2 Department of Medicine, School of Medicine, Pontificia Universidade Catolica do Parana, Curitiba, Brazil; 3 Arbor Research Collaborative for Health, Ann Arbor, Michigan; 4 Global Medical Office, Fresenius Medical Care, Buenos Aires, Argentina; 5 Department of Medicine, Escuela de Medicina y Ciencias de la Salud, Tecnologico de Monterrey, Monterrey, Mexico; 6 Department of Medicine, Hospital Zambrano Hellion, TecSalud, Instituto de Medicina Interna, Tecnologico de Monterrey, San Pedro Garza García, Mexico; 7 Departmento de Clínicas Médicas, Centro Universitario de Ciencias de la Salud, Universidad de Guadalajara, Guadalajara, Mexico; 8 Renal Research Institute, New York, New York; 9 Science Department, Katz School of Science and Health at Yeshiva University, New York, New York; 10 Department of Information Systems and Operations Management, Tilburg University, Tilburg, The Netherlands; 11 Department of Internal Medicine, Catharina Hospital Eindhoven, Eindhoven, The Netherlands

**Keywords:** creatinine clearance, nutrition, peritoneal dialysis, phosphate binders

## Abstract

**Key Points:**

- This is a retrospective observational multinational peritoneal dialysis study to test whether creatinine clearance could be a better marker of serum phosphorus than urea Kt/V.
- Creatinine clearance was not more accurate predicting serum phosphorus than urea Kt/V, but its inclusion in multivariable models added more clarity.
- In conclusion, using both biomarkers, instead of just one, may better assist in the optimization of serum phosphorus levels.

**Background:**

Hyperphosphatemia is associated with poor outcome and is still very common in peritoneal dialysis (PD) patients. Because peritoneal phosphorus clearance is closer to peritoneal creatinine clearance (CrCl) than urea clearance, we hypothesized that weekly CrCl could be a better marker of serum phosphorus in PD.

**Methods:**

In a retrospective observational study, data from adult PD patients were collected across five institutions in North and South America: Fresenius Medical Care Latin America, Renal Research Institute, Mount Sinai Hospital, Hospital Civil de Guadalajara, and the Brazil PD cohort. All centers analyzed routinely available laboratory data, with exclusions for missing data on serum phosphorus, CrCl, or urea Kt/V. A unified statistical protocol was used across centers. Linear mixed-effect models examined associations between longitudinal serum phosphorus levels, CrCl, and Kt/V. Adjustments were made for age, sex, and baseline phosphorus binder usage. Mixed-effects meta-analysis determined the pooled effect size of CrCl and Kt/V on serum phosphorus trajectories, adjusted for confounders.

**Results:**

There were 16,796 incident PD patients analyzed. Age, body mass index, sex, PD modality, Kt/V, and CrCl, as well as serum phosphorus, varied significantly across the different cohorts, but >70% had residual renal function. For most cohorts, both CrCl total and urea Kt/V associated negatively with serum phosphorus levels, and log-likelihood ratio tests demonstrate that models including CrCltotal have more predictive information than those including only urea Kt/V for the largest cohorts. Models including CrCltotal increase information predicting longitudinal serum phosphorus levels irrespective of baseline urea Kt/V, age, use of phosphorus binder, and sex.

**Conclusions:**

CrCl was not more accurate in predicting serum phosphorus than urea Kt/V, but its inclusion in multivariable models predicting serum phosphorus added accuracy. In conclusion, both CrCl and Kt/V are associated with phosphorus levels, and using both biomarkers, instead of just one, may better assist in the optimization of serum phosphorus levels.

## Visual Abstract

**Figure:**
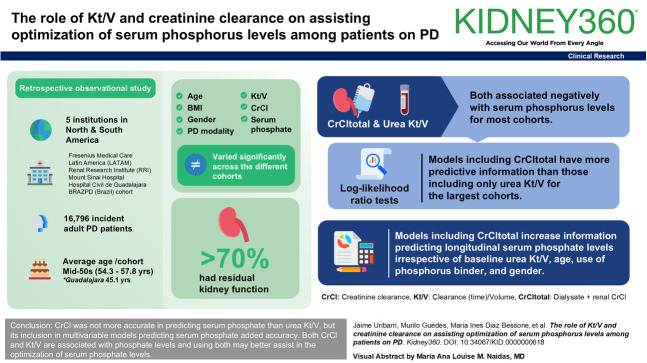

## Introduction

Guidelines on the management of peritoneal dialysis (PD) patients have undergone substantial changes over the past decades.^1,2^ Initially, an approach mainly focused on the clearance of small molecules (namely Kt/V) was recommended; currently, a more comprehensive, holistic view that involves various clinical, quality of life, and laboratory parameters is recommended. This was particularly emphasized in a recent publication by the International Society of PD, which aimed to provide guidance on how to most optimally provide PD.^1^ During this change in mindset, monitoring of several additional parameters by the renal care team have been added. The most recent International Society of PD guideline includes more than 14 domains, with each of them covering numerous assessments, such as serum phosphorus, creatinine clearance (CrCl), and urea Kt/V.

The guideline also clearly expressed concerns about the feasibility of such detailed assessments, particularly in low- and middle-income countries. For this reason, studies on the parallels between some biomarkers could be useful to reduce the scope of assessments to a subset of essential markers, facilitating optimal care in an environment with limited resources.

Phosphorus is a small molecule with clearance characteristics comparable with a medium-sized molecule when hydrated and is essential to be measured as a marker of mineral bone health but is also associated with administered dialysis dose and nutrition.^3–5^

The dimensionless Kt/V urea measures the removal of urea, as a representative measure of small solute removal by either renal function of renal replacement therapy, while CrCl measures the removal of creatinine as a representative of larger molecules. Which of those two markers most closely reflects phosphorus removal has not been entirely understood as per the currently published literature. We aimed to investigate the association between CrCl and Kt/V urea with serum phosphorus levels in a study of longitudinal data of International PD cohorts.

## Methods

### Study Design

We designed and conducted a retrospective observational study of longitudinal data of international PD cohorts from different partnering institutions. Data were analyzed by the respective institutions and then analyzed in aggregation. Data from Latin America (LATAM) includes patients starting PD between January 2000 until December 2021. However, laboratory data were fully available starting October 2002 (CrCl) and December 2003 (phosphorus). In each center, patient follow-up started at PD initiation and ended at transfer to another facility, modality change (*e.g*., transplant or hemodialysis), lost to follow-up, or death. All participating centers routinely analyzed available laboratory data during the study follow-up period. Patients without information on serum phosphorus, CrCl, and Kt/V were excluded from the analysis. Each study cohort got its own local Institutional Review Board approval with no individual consent form signed, except within the Brazil PD cohort (BRAZPD), since data collection was part of the routine clinical care of these patients.

### Data Sources

We included data of adult patient older than 18 years from five different partner institutions throughout the United States, Mexico, and South America: Fresenius Medical Care (FMC-LATAM; LATAM), Renal Research Institute (RRI; United States), Mount Sinai Hospital (New York, United States), Hospital Civil de Guadalajara Fray Antonio Alcalde (Guadalajara, Mexico), and the BRAZPD (Brazil) cohort.

In short, the BRAZPD cohort was a prospective observational study during 2004–2011 that captured routinely available data from several clinics in Brazil. Data from Hospital Civil de Guadalajara was collected between 2017 and 2021 and was curated by medical professionals, including doctors and nurses, specifically designed to support the Hospital Civil PD Program. Data from Mount Sinai Hospital were collected between 2007 and 2021 from electronic health records in the institution. Data from LATAM and RRI were abstracted from a central database of electronic health records of PD patients in LATAM and the United States.

### Variables

We designed and conducted a retrospective observational study of longitudinal data of international PD cohorts from different partnering institutions. Data were analyzed by the respective institutions and then analyzed in aggregation. In each center, patient follow-up started at PD initiation and ended at transfer to another facility, modality change (*e.g*., transplant or hemodialysis), lost to follow-up, or death. All participating centers routinely analyzed available laboratory data during the study follow-up period. Patients without information on serum phosphorus, CrCl, and Kt/V were excluded from the analysis. Each study cohort got its own local Institutional Review Board approval with no individual consent form signed, except within the BRAZPD cohort, since data collection was part of the routine clinical care of these patients.^6^

Daily urea clearance (K) was estimated in L/d by the general clearance formula: (dialysate urea concentration×dialysis daily output)/plasma urea concentration, paying attention to units so final K is in L/d. The same formula was used to calculate renal urea clearance, only changing the terms from dialysate to urine.

Nitrogen appearance rate (nPCR) was calculated from the sum of values in dialysate and urine using the Randerson equation: nPCR (g/d)=10.76 (urea nitrogen generation in mg/min+1.46)/(V/0.58).^7^ V in this formula is the total body water calculated by the Watson equation.^6^

Residual kidney function (RKF) was defined as the presence of a urinary output >100 ml/d.

Weekly CrCl was calculated by estimating daily CrCl (dialysate, renal, or both) multiplied by seven. Daily dialysate CrCl was estimated by the general clearance formula: (dialysate creatinine concentration×dialysis daily output)/plasma creatinine concentration, paying attention to units so final value is in L/d. The same formula was used to calculate renal CrCl, only changing the terms from dialysate to urine.

Our primary objective was to estimate the association between serum phosphorus levels and baseline CrCl and Kt/V, adjusted for key confounders. Because serum phosphorus is routinely collected in PD care, we extracted all serum phosphorus levels over time during the study follow-up.

### Statistical Methods

Patient characteristics are presented as means±SD or medians with interquartile ranges, contingent on the data distribution. To estimate the associations between longitudinal serum phosphorus levels and both creatinine CrCl and Kt/V, we used linear mixed-effect models. These models designated baseline CrCl and Kt/V as fixed effects while incorporating patient-specific random intercepts and slopes. Adjustments were made for age, sex, and baseline phosphorus binder usage. We further conducted subgroup analyses stratified by RKF, PD modality, and initial phosphorus binder use. To ascertain whether models integrating both CrCl and Kt/V bolstered serum phosphorus prediction compared with those using Kt/V alone, we applied log-likelihood ratio (LLR) tests with a significance threshold set at *α*=0.05. Subsequently, a mixed-effects meta-analysis was used to derive the pooled effect size concerning the associations between CrCl and Kt/V on serum phosphorus trajectories. This effect size was adjusted for the aforementioned covariates. Pooled unstandardized linear coefficients were computed using the inverse variance method, and the 95% confidence intervals were ascertained employing the DerSimonian and Laird approach.

## Statement of Ethics

### Study Approval Statement

There were five study cohorts:

- Fresenius Medical Care LATAM. They used data collected during the provision of routine medical care in dialysis patients. All data were deidentified for the purposes of this study analyses. The Europeanl Clinical Database (EuClid) database was used for capturing data in the LATAM cohort as part of Fresenius Medical Care's quality improvement and management programs in all NephroCare clinics. EuCLiD governance has established protocols and procedures for use of clinical data from NephroCare clinics for secondary research purposes. Data were only collected from patients who provided informed consent for their data to be collected into EuCliD, and the data were deidentified by the LATAM investigator. The analysis in each region was conducted in accordance with the Declaration of Helsinki.
- RRI (New York). Renal Research Institute uses The Western Institutional Review Board that deemed this study as exempt from board review.
- Mount Sinai Hospital (New York). This study was reviewed and approved by the Icahn School of Medicine at Mount Sinai Institutional Review Board (IRB 19–01467) with exemption of consent.
- Hospital Civil de Guadalajara Fray Antonio Alcalde (Guadalajara, Mexico). The study was conducted in accordance with the World Medical Association Declaration of Helsinki. This study was exempted from ethical committee approval by the Hospital Civil de Guadalajara Fray Antonio Alcalde. Informed consent was waived because data were analyzed as a deidentified dataset.
- BRAZPD (Brazil) cohort. The observational cohort data from BRAZPD were collected prospectively in a deidentified manner, following a protocol that received approval from the Institutional Review Board of Pontificia Universidade Catolica do Parana (Curitibia, Parana, Brazil; approval number 25000.187284/2004–1) with informed consent obtained from all patients.

### Consent to Participate Statement

Witten informed consent for participation was only obtained in the BRAZPD cohort. The need for informed consent was waived in all other cohorts.

## Results

A total of 16,796 patients were included in this analysis, with a majority from LATAM (94%). Table 1 describes patients' main demographic characteristics and selected study parameters throughout the different cohorts. The average age across the cohorts was mid-late 50s, except for 45.1 years in Guadalajara. Gender distribution showed relative uniformity, with male patients making up about half of each cohort; the most notable difference was observed in the Guadalajara cohort, where 62.9% of patients were male.

**Table 1:** Demographics and average selected study parameters across the different cohorts

Cohort	RRI	Sinai	BRAZPD	FMC-LATAM	Guadalajara
*N*	653	131	171	15,631	210
Age (yr)	55.7±16.3	57.8±15.5	54.3±14.4	56.5±16.5	45.1±17.3
Male (%)	362 (55.4)	71 (54.2)	86 (50.3)	8172 (52.3)	132 (62.9)
APD (%)	343 (52.5)	122 (93.1)	108 (63.2)	2699 (17.3)	16 (8.0)
CAPD (%)	310 (47.5)	8 (6.1)	59 (34.5)	11,253 (72.0)	194 (92.0)
RKF (%)	527 (80.7)	92 (70.2)	165 (96.5)	11,090 (70.9)	151 (71.9)
urea Kt/V_total_	2.17±0.6	2.1±0.7	2.5±0.9	2.4±0.7	1.7±0.5
CrCl_total_ (ml/min per 1.73 m^2^)	103.7±52.8	76.7±46.5	96.3±49.4	74.2±39.0	63.8±31.3
Serum PO_4_ (mg/dl)	5.7±1.8	5.6±1.6	5.0±1.5	4.9±1.3	5.8±1.9
P-binders use (%)	518 (79.3)	96 (73.3)	115 (67.3)	7034 (45.0)	41 (19.5)
nPCR (g/kg per day)	1.0±0.3	0.74±0.2	1.3±0.4	0.8±0.3	1.1±0.3

Parameters expressed as mean±SD unless otherwise specified. APD, automatic peritoneal dialysis; BRAZPD, Brazil PD; CAPD, continuous ambulatory peritoneal dialysis; CrCl, creatinine clearance; FMC-LATAM, Fresenius Medical Care-Latin America; Guadalajara, Hospital Civil de Guadalajara Fray Antonio Alcalde; PO, serum phosphorus; nPCR, nitrogen appearance rate; RKF, residual kidney function (urinary output >100 ml/d); RRI, Renal Research Institute; Sinai, mount sinai hospital.

Dialysis modality distribution varied quite substantially; automatic PD was the prevalent modality in most cohorts (52.5% in RRI, 93.1% in Sinai, 63.2% in BRAZPD), except in FMC-LATAM, in which continuous ambulatory PD was more common (72%).

The proportion of patients with RKF was higher than 70% in all cohorts. Weekly urea Kt/V values across cohorts were fairly similar, with a slight dip in the Guadalajara cohort (1.7±0.5). Weekly CrCl total (dialysate+renal) varied significantly across cohorts, with the RRI cohort displaying the highest mean (103.7±52.8 L/w) and Guadalajara the lowest (63.8±31.3 L/w).

**Figure 1:**
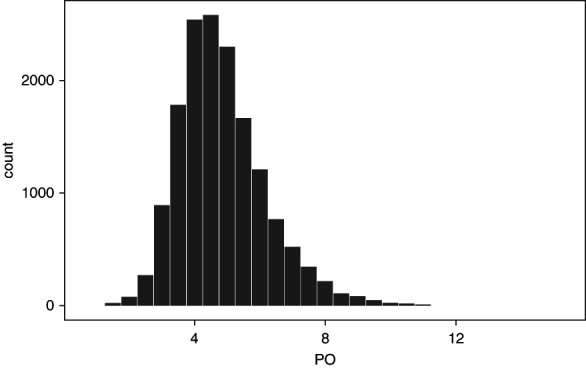
**Distribution of serum phosphorus levels in the FMC-LATAM cohort.** *x* axis: serum phosphorus levels expressed in mg/dl; *y* axis: patients count. FMC-LATAM, Fresenius Medical Care-Latin America; PO, serum phosphorus.

Serum phosphorus concentrations and the use of phosphorus binders varied across the different cohorts, with the FMC-LATAM and BRAZPD cohorts having the lowest serum phosphorus level (4.9±1.3 and 5.0±1.5 mg/dl, respectively), and the RRI cohort showing the highest percent of phosphorus binder use (79.3%). The variability of serum phosphorus levels in the largest cohort (FMC-LATAM) is provided in Figure 1. Histograms of serum phosphorus distributions from other participating sites are reported in Supplemental Figure 1.

The nPCR, a marker of dietary protein intake in metabolically stable patients, exhibited a broad range across cohorts. The BRAZPD cohort had the highest nPCR (1.3±0.4 g/d per kilogram), whereas the Sinai cohort showed the lowest (0.74±0.2 g/d per kilogram).

The results of the cross-sectional associations between serum phosphorus, CrCl total, and urea Kt/V are described in Table 2. For most cohorts, both CrCl total and urea Kt/V associated negatively with serum phosphorus levels, and LLR demonstrate that models including CrCltotal have more predictive information than those including only urea Kt/V for the largest cohorts (RRI and FMC-LATAM). The effect estimates in Table 2 represent the linear relationship between serum phosphorus and Kt/V and CrCl, respectively. These results represent the change in serum phosphorus by a unit change in the exposure variables. Because the scales of Kt/V and CrCl are different, the magnitude of the strength of the association must be interpreted with caution when comparing Kt/V and CrCl results.

**Table 2:** Cross-sectional associations between serum phosphorus, creatinine clearance, and urea Kt/V

Cohorts	Serum PO_4_ and CrCl			Serum PO_4_ and Kt/V		
	Estimate	Lower CI	Upper CI	Estimate	Lower CI	Upper CI
RRI	−0.015	−0.018	−0.012	−1.437	−1.721	−1.152
Sinai	−0.009	−0.016	−0.003	−0.906	−1.410	−0.403
BRAZPD	−0.008	−0.012	−0.003	−0.735	−1.105	−0.366
FMC-LATAM	−0.010	−0.010	−0.009	−0.589	−0.618	−0.560
Guadalajara	0.002	−0.005	0.010	−0.220	−0.840	0.380

Comparison of nested models including creatinine clearance with those not including creatinine clearance, adjusting for serum Kt/V, age, sex, and use of phosphorus binders. The CI provides the 95% values. *P* values lower than 0.05 rule out the null hypothesis of no information added by creatinine clearance. BRAZPD, Brazil PD; CI, confidence interval; CrCl, creatinine clearance; FMC-LATAM, Fresenius Medical Care-Latin America; PO, serum phosphorus; RRI, Renal Research Institute.

**Figure 2:**
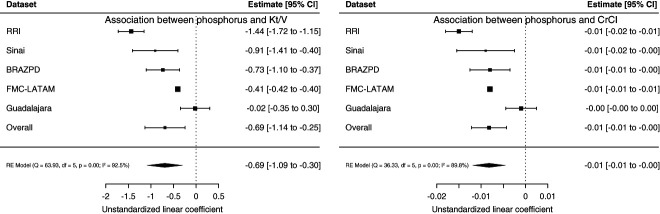
**Forest plot of the associations between longitudinal serum phosphorus and Kt/V and CrCl.** Estimates with CI and statistical significance for the association between longitudinal serum phosphorus and Kt/V (left panel) and CrCl (right panel) for each of the cohorts as well as overall. BRAZPD, Brazil PD; CI, confidence interval; CrCl, creatinine clearance RE, random effects; RRI, Renal Research Institute.

As summarized in Figure 2, longitudinal phosphorus levels are associated with baseline CrCl total and urea Kt/V in nearly all participating cohorts. Pooled unstandardized linear coefficients confirm negative associations between CrCl total, urea Kt/V, and serum phosphorus, although at a high heterogeneity level (I^2^=92.5% for Kt/V and 89.8% for CrCl total). LLR for models in each cohort yielded *P* values consistently lower than 0.05, confirming that models including CrCltotal increase information to predict longitudinal serum phosphorus levels irrespective of baseline urea Kt/V, age, use of phosphorus binder, and sex.

The distribution of Kt/V per CrCl by serum phosphorus levels in the different cohorts are depicted in Supplemental Figures 2–6.

## Discussion

In this multinational, multicenter study of incident PD patients, both weekly urea Kt/V and weekly CrCl were associated with serum phosphorus levels, both cross-sectionally and longitudinally. However, we could not find superiority of weekly CrCl in predicting serum phosphorus, contrary to our expectations on the basis of the similar molecular size of creatinine and hydrated phosphorus. Our study demonstrates the complexity in understanding and implementing control and management of a traditional biomarker, like phosphorus, in dialysis patients. First, we sought to characterize our population, and their serum phosphorus levels, and, right from the start, we observed a great variability in phosphorus values across different cohorts. We are aware that phosphorus serum levels in a dialysis patient depend on multiple factors, including diet, the presence of residual diuresis, the patient's membrane profile, the modality of PD and how it is prescribed, as well as the use of different phosphorus binders. As such, we conclude that a multifactorial assessment of all dimension remains indicated.

The prescription of phosphorus binders in our cohort is representative of the practice observed worldwide. For example, the two North American cohorts had a percentage of patients using binders around 70%, similar to the values reported by the United States Renal Data System, where in 2020 approximately 62% of PD patients were taking one or more types of binders in the United States.^8^ Similar to our database, the vast majority of large registries lack detailed dietary data. Therefore, we sought to indirectly assess nPCR estimates from the urea kinetics model as a marker of protein intake, hoping to aid in predicting serum phosphorus levels. Like others who have preceded us, we found a significant association between nPCR and serum phosphorus. However, despite its statistical significance, there was again considerable variability in the association.^9^

Serum phosphorus levels are inversely correlated with peritoneal phosphorus clearance, and several prior studies have shown that dialysis phosphorus clearance is comparable with dialysis CrCl.^10,11^ On the other hand, for decades, the dominant concept in PD has been a therapy control primarily centered around urea. Although the importance of Kt/V has been recently downgraded in patient management, it still remains within the guidelines.

It is understandable and natural for our concepts to change over time, and guidelines in PD have indeed evolved. However, the current complexity is substantial, and for countries with low to medium socioeconomic levels, strategies to reduce costs are particularly important. In a systematic review from 2015, despite the heterogeneity in how different authors measure dialysis costs worldwide, there is a consensus that the identified costs significantly exceed the local financial capacity.^12^ Our goal was to determine whether two classic PD tools, CrCl and KtV, could somehow be prioritized or de-emphasized in making decisions about controlling serum phosphorus. Despite the negative (and statistically significant) correlation between these measures and serum phosphorus levels, the large heterogeneity observed reflects the scientific community's challenge in defining clear markers or therapeutic targets for a more practical management of PD.

This study has several limitations. First, these data were not collected as part of a prospectively designed study, but restricted (for the most part) to data collected from routine clinical practice. Because practice patterns change through different countries not all variables possibly of interest for our analyses were available for all cohorts. Because of an unevenly distributed number of patients and also parameters measured in each patient among the partnering institutions, interpretation was more complex, and we were not able to conduct analyses in all cohort equally. As a result, the adjustments did not include all possibly relevant factors. Given the high level of heterogeneity between the cohorts and the analytic meta-analysis approach, estimates are also presenting with a larger level of variability, as may have been observed with a combined dataset. Another limitation of our study is the lack of temporal data on Kt/V, membrane profile, and RKF measurements in the dataset from most contributing partner institutions. The temporal relationship between these variables is important because of their influence on solute removal. However, these data were not routinely collected, and only a few records include them. Furthermore, we restricted our study to incident PD patients who notably had substantial residual renal clearance, which may result in variance to estimates seen in a cohort of prevalent PD patients. The results of Kt/V for the entire population are based on estimates of total body water using traditional approaches. However, we are aware that significant intraindividual variations have been reported recently, and this could potentially interfere with the interpretation of our results.^13^

A few more words about our methods are necessary. The main focus and more robust estimates were obtained from an linear mixed-effect model, making it superior to the initial cross-sectional analysis for several reasons. First, a linear mixed-effect model allows us to account for both fixed and random effects, enabling us to consider individual variations and the hierarchical structure of the data, such as differences between centers. This leads to more precise and reliable estimates. Second, LMMs can handle missing data more effectively and provide more accurate standard errors, which are critical for valid inference and clinical decision-making. The ability to include random effects allows us to control for unobserved heterogeneity, which is often present in clinical data. Finally, a cross-sectional analysis does not account for the inherent variability and correlation within the data, potentially leading to biased estimates.

In conclusion, both CrCl and Kt/V are associated with phosphorus levels, and using both biomarkers, instead of just one, may better assist in the optimization of serum phosphorus levels among patients on PD.

## Supplementary Material

## Data Availability

All data are included in the manuscript and/or supporting information.

## Supplemental Material

Supplemental Figure 1. Distribution of serum phosphate levels in the cohorts other than LATAM.

Supplemental Figures 2–6. Different plots phosphorus versus Kt/V and CrCl across the different cohorts.
