# Supplementary material for: The Role of Kt/V and Creatinine Clearance on Assisting Optimization of Serum Phosphorus Levels among Patients on Peritoneal Dialysis
Source: Kidney360. 2024 Oct 11;6(1):105–11. doi: 10.34067/KID.0000000618 (PMC11793178; doi:10.34067/KID.0000000618)
Supplement: Supplementary file 1 [file kidney360-6-105-s001.pdf]

ASN American Society of Nephrology · JASN Journal of the American Society of Nephrology · KIDNEY360 Accessing Our World From Every Angle · CJASN Clinical Journal of the American Society of Nephrology

ASN Journal Disclosure Form

As per ASN journal policy, I have disclosed any financial relationships or commitments I have held in the past 36 months as included below. I have listed my Current Employer below to indicate there is a relationship requiring disclosure. If no relationship exists, my Current Employer is not listed.

L. Chan reports the following:
Employer: Icahn School of Medicine at Mount Sinai; Consultancy: CSL Vifor Pharma, INC; and Research Funding: NIH.

I understand that the information above will be published within the journal article, if accepted, and that failure to comply and/or to accurately and completely report the potential financial conflicts of interest could lead to the following: 1) Prior to publication, article rejection, or 2) Post-publication, sanctions ranging from, but not limited to, issuing a correction, reporting the inaccurate information to the authors' institution, banning authors from submitting work to ASN journals for varying lengths of time, and/or retraction of the published work.

Name: Lili Chan
Manuscript ID: K360-2024-000265R2
Manuscript Title: The role of Kt/V and creatinine clearance on assisting optimization of serum phosphorus levels among patients on PD
Date of Completion: August 26, 2024
Disclosure Updated Date: May 8, 2024

ASN American Society of Nephrology | JASN Journal of the American Society of Nephrology | KIDNEY360 Accessing Our World From Every Angle | CJASN Clinical Journal of the American Society of Nephrology

ASN Journal Disclosure Form

As per ASN journal policy, I have disclosed any financial relationships or commitments I have held in the past 36 months as included below. I have listed my Current Employer below to indicate there is a relationship requiring disclosure. If no relationship exists, my Current Employer is not listed.

A. De la Torre Quiroga reports the following:
Employer: ITESM

I understand that the information above will be published within the journal article, if accepted, and that failure to comply and/or to accurately and completely report the potential financial conflicts of interest could lead to the following: 1) Prior to publication, article rejection, or 2) Post-publication, sanctions ranging from, but not limited to, issuing a correction, reporting the inaccurate information to the authors' institution, banning authors from submitting work to ASN journals for varying lengths of time, and/or retraction of the published work.

Name: Andres E. De la Torre Quiroga
Manuscript ID: K360-2024-000265R2
Manuscript Title: The role of Kt/V and creatinine clearance on assisting optimization of serum phosphorus levels among patients on PD.
Date of Completion: August 26, 2024
Disclosure Updated Date: August 26, 2024

ASN
American Society of Nephrology
JASN
Journal of the American Society of Nephrology
KIDNEY360
Accessing Our World From Every Angle
CJASN
Clinical Journal of the American Society of Nephrology

ASN Journal Disclosure Form

As per ASN journal policy, I have disclosed any financial relationships or commitments I have held in the past 36 months as included below. I have listed my Current Employer below to indicate there is a relationship requiring disclosure. If no relationship exists, my Current Employer is not listed.

M. Diaz Bessone reports the following:
Employer: Fresenius Medical Care

I understand that the information above will be published within the journal article, if accepted, and that failure to comply and/or to accurately and completely report the potential financial conflicts of interest could lead to the following: 1) Prior to publication, article rejection, or 2) Post-publication, sanctions ranging from, but not limited to, issuing a correction, reporting the inaccurate information to the authors' institution, banning authors from submitting work to ASN journals for varying lengths of time, and/or retraction of the published work.

Name: Maria Ines Diaz Bessone
Manuscript ID: K360-2024-000265R2
Manuscript Title: The role of Kt/V and creatinine clearance on assisting optimization of serum phosphorus levels among patients on PD.
Date of Completion: October 1, 2024
Disclosure Updated Date: October 1, 2024

ASN American Society of Nephrology JASN Journal of the American Society of Nephrology KIDNEY360 Accessing Our World From Every Angle CJASN Clinical Journal of the American Society of Nephrology

ASN Journal Disclosure Form

As per ASN journal policy, I have disclosed any financial relationships or commitments I have held in the past 36 months as included below. I have listed my Current Employer below to indicate there is a relationship requiring disclosure. If no relationship exists, my Current Employer is not listed.

D. Farrell reports the following:
Employer: Icahn School of Medicine at Mount Sinai

I understand that the information above will be published within the journal article, if accepted, and that failure to comply and/or to accurately and completely report the potential financial conflicts of interest could lead to the following: 1) Prior to publication, article rejection, or 2) Post-publication, sanctions ranging from, but not limited to, issuing a correction, reporting the inaccurate information to the authors' institution, banning authors from submitting work to ASN journals for varying lengths of time, and/or retraction of the published work.

Name: Douglas R. Farrell
Manuscript ID: K360-2024-000265R2
Manuscript Title: The role of Kt/V and creatinine clearance on assisting optimization of serum phosphorus levels among patients on PD.
Date of Completion: September 26, 2024
Disclosure Updated Date: September 26, 2024

ASN American Society of Nephrology | JASN Journal of the American Society of Nephrology | KIDNEY360 Accessing Our World From Every Angle | CJASN Clinical Journal of the American Society of Nephrology

ASN Journal Disclosure Form

As per ASN journal policy, I have disclosed any financial relationships or commitments I have held in the past 36 months as included below. I have listed my Current Employer below to indicate there is a relationship requiring disclosure. If no relationship exists, my Current Employer is not listed.

G. Garcia-Garcia reports the following:
Employer: University of Guadalajara Health Sciences Center; Consultancy: Ellen Medical Devices, LTD; Patents or Royalties: Elsevier Inc.; and Other Interests or Relationships: Associate editor, CJASN.

I understand that the information above will be published within the journal article, if accepted, and that failure to comply and/or to accurately and completely report the potential financial conflicts of interest could lead to the following: 1) Prior to publication, article rejection, or 2) Post-publication, sanctions ranging from, but not limited to, issuing a correction, reporting the inaccurate information to the authors' institution, banning authors from submitting work to ASN journals for varying lengths of time, and/or retraction of the published work.

Name: Guillermo Garcia-Garcia
Manuscript ID: K360-2024-000265R1
Manuscript Title: "The role of Kt/V and creatinine clearance on assisting optimization of serum phosphorus levels among patients on PD.,"
Date of Completion: July 25, 2024
Disclosure Updated Date: May 7, 2024

ASN American Society of Nephrology | JASN Journal of the American Society of Nephrology | KIDNEY360 Accessing Our World From Every Angle | CJASN Clinical Journal of the American Society of Nephrology

ASN Journal Disclosure Form

As per ASN journal policy, I have disclosed any financial relationships or commitments I have held in the past 36 months as included below. I have listed my Current Employer below to indicate there is a relationship requiring disclosure. If no relationship exists, my Current Employer is not listed.

M. Guedes reports the following:
Research Funding: AstraZeneca; and Honoraria: Astrazeneca.

I understand that the information above will be published within the journal article, if accepted, and that failure to comply and/or to accurately and completely report the potential financial conflicts of interest could lead to the following: 1) Prior to publication, article rejection, or 2) Post-publication, sanctions ranging from, but not limited to, issuing a correction, reporting the inaccurate information to the authors' institution, banning authors from submitting work to ASN journals for varying lengths of time, and/or retraction of the published work.

Name: Murilo Henrique Guedes
Manuscript ID: K360-2024-000265R2
Manuscript Title: The role of Kt/V and creatinine clearance on assisting optimization of serum phosphorus levels among patients on PD.
Date of Completion: September 26, 2024
Disclosure Updated Date: September 26, 2024

ASN
American Society of Nephrology
JASN
Journal of the American Society of Nephrology
KIDNEY360
Accessing Our World From Every Angle
CJASN
Clinical Journal of the American Society of Nephrology

ASN Journal Disclosure Form

As per ASN journal policy, I have disclosed any financial relationships or commitments I have held in the past 36 months as included below. I have listed my Current Employer below to indicate there is a relationship requiring disclosure. If no relationship exists, my Current Employer is not listed.

A. Guinsburg reports the following:
Employer: Fresenius Medical Care; and Advisory or Leadership Role: Chief Clinical Officer Fresenius Medical Care LatAm.

I understand that the information above will be published within the journal article, if accepted, and that failure to comply and/or to accurately and completely report the potential financial conflicts of interest could lead to the following: 1) Prior to publication, article rejection, or 2) Post-publication, sanctions ranging from, but not limited to, issuing a correction, reporting the inaccurate information to the authors' institution, banning authors from submitting work to ASN journals for varying lengths of time, and/or retraction of the published work.

Name: Adrian M. Guinsburg
Manuscript ID: K360-2024-000265R1
Manuscript Title: The role of Kt/V and creatinine clearance on assisting optimization of serum phosphorus levels among patients on PD
Date of Completion: July 25, 2024
Disclosure Updated Date: May 16, 2024

ASN Journal Disclosure Form

As per ASN journal policy, I have disclosed any financial relationships or commitments I have held in the past 36 months as included below. I have listed my Current Employer below to indicate there is a relationship requiring disclosure. If no relationship exists, my Current Employer is not listed.

C. Konings reports the following:
Employer: Catharina Hospital Eindhoven Holland

I understand that the information above will be published within the journal article, if accepted, and that failure to comply and/or to accurately and completely report the potential financial conflicts of interest could lead to the following: 1) Prior to publication, article rejection, or 2) Post-publication, sanctions ranging from, but not limited to, issuing a correction, reporting the inaccurate information to the authors' institution, banning authors from submitting work to ASN journals for varying lengths of time, and/or retraction of the published work.

Name: Constantijn Konings
Manuscript ID: K360-2024-000265R2
Manuscript Title: The role of Kt/V and creatinine clearance on assisting optimization of serum phosphorus levels among patients on PD
Date of Completion: August 26, 2024
Disclosure Updated Date: May 14, 2024

ASN American Society of Nephrology
JASN Journal of the American Society of Nephrology
KIDNEY360 Accessing Our World From Every Angle
CJASN Clinical Journal of the American Society of Nephrology

ASN Journal Disclosure Form

As per ASN journal policy, I have disclosed any financial relationships or commitments I have held in the past 36 months as included below. I have listed my Current Employer below to indicate there is a relationship requiring disclosure. If no relationship exists, my Current Employer is not listed.

P. Kotanko reports the following:
Employer: Renal Research Institute; Ownership Interest: Fresenius Medical Care; Research Funding: Fresenius Medical Care; NIH; KidneyX; PCORI; Patents or Royalties: Multiple patents in the kidney space; and Advisory or Leadership Role: Editorial Board of Blood Purification; Editorial Board of Kidney and Blood Pressure Research; Editorial Board of Frontiers in Nephrology.

I understand that the information above will be published within the journal article, if accepted, and that failure to comply and/or to accurately and completely report the potential financial conflicts of interest could lead to the following: 1) Prior to publication, article rejection, or 2) Post-publication, sanctions ranging from, but not limited to, issuing a correction, reporting the inaccurate information to the authors' institution, banning authors from submitting work to ASN journals for varying lengths of time, and/or retraction of the published work.

Name: Peter Kotanko
Manuscript ID: K360-2024-000265R2
Manuscript Title: The role of Kt/V and creatinine clearance on assisting optimization of serum phosphorus levels among patients on PD
Date of Completion: September 8, 2024
Disclosure Updated Date: May 6, 2024

ASN American Society of Nephrology | JASN Journal of the American Society of Nephrology | KIDNEY360 Accessing Our World From Every Angle | CJASN Clinical Journal of the American Society of Nephrology

ASN Journal Disclosure Form

As per ASN journal policy, I have disclosed any financial relationships or commitments I have held in the past 36 months as included below. I have listed my Current Employer below to indicate there is a relationship requiring disclosure. If no relationship exists, my Current Employer is not listed.

A. Mermelstein reports the following:
Employer: Renal Research Institute; and Research Funding: Vifor Fresenius Medical Care Renal Pharma.

I understand that the information above will be published within the journal article, if accepted, and that failure to comply and/or to accurately and completely report the potential financial conflicts of interest could lead to the following: 1) Prior to publication, article rejection, or 2) Post-publication, sanctions ranging from, but not limited to, issuing a correction, reporting the inaccurate information to the authors' institution, banning authors from submitting work to ASN journals for varying lengths of time, and/or retraction of the published work.

Name: Ariella E. Mermelstein
Manuscript ID: K360-2024-000265R1
Manuscript Title: The role of Kt/V and creatinine clearance on assisting optimization of serum phosphorus levels among patients on PD.
Date of Completion: July 25, 2024
Disclosure Updated Date: May 28, 2024

ASN
American Society of Nephrology
JASN
Journal of the American Society of Nephrology
KIDNEY360
Accessing Our World From Every Angle
CJASN
Clinical Journal of the American Society of Nephrology

ASN Journal Disclosure Form

As per ASN journal policy, I have disclosed any financial relationships or commitments I have held in the past 36 months as included below. I have listed my Current Employer below to indicate there is a relationship requiring disclosure. If no relationship exists, my Current Employer is not listed.

T. Moraes reports the following:
Employer: Pontificia Universidade Catolica do Parana; Consultancy: Astrazeneca; Baxter Healthcare; Bayer; Boehringer; Lilly; Novo Nordisk; Research Funding: Baxter Healthcare; Honoraria: Astrazeneca; Baxter Healthcare; Bayer; Boehringer; Lilly; Novo Nordisk; and Advisory or Leadership Role: Peritoneal Dialysis International - Editorial Board; Brazilian Journal of Nephrology - Co-Editor in Chief; International Society for Peritoneal Dialysis - Coordinator ISPD International Studies Committee.; Latin American Society of Nephrology and Hypertension (SLANH) - Coordinator Peritoneal Dialysis Comittee.

I understand that the information above will be published within the journal article, if accepted, and that failure to comply and/or to accurately and completely report the potential financial conflicts of interest could lead to the following: 1) Prior to publication, article rejection, or 2) Post-publication, sanctions ranging from, but not limited to, issuing a correction, reporting the inaccurate information to the authors' institution, banning authors from submitting work to ASN journals for varying lengths of time, and/or retraction of the published work.

Name: Thyago Proença de Moraes
Manuscript ID: K360-2024-000265R2
Manuscript Title: The role of Kt/V and creatinine clearance on assisting optimization of serum phosphorus levels among patients on PD.
Date of Completion: August 30, 2024
Disclosure Updated Date: May 14, 2024

ASN American Society of Nephrology JASN Journal of the American Society of Nephrology KIDNEY360 Accessing Our World From Every Angle CJASN Clinical Journal of the American Society of Nephrology

ASN Journal Disclosure Form

As per ASN journal policy, I have disclosed any financial relationships or commitments I have held in the past 36 months as included below. I have listed my Current Employer below to indicate there is a relationship requiring disclosure. If no relationship exists, my Current Employer is not listed.

V. Peters reports the following:
Employer: Tilburg University

I understand that the information above will be published within the journal article, if accepted, and that failure to comply and/or to accurately and completely report the potential financial conflicts of interest could lead to the following: 1) Prior to publication, article rejection, or 2) Post-publication, sanctions ranging from, but not limited to, issuing a correction, reporting the inaccurate information to the authors' institution, banning authors from submitting work to ASN journals for varying lengths of time, and/or retraction of the published work.

Name: Vincent Peters
Manuscript ID: K360-2024-000265R1
Manuscript Title: The role of Kt/V and creatinine clearance on assisting optimization of serum phosphorus levels among patients on PD.
Date of Completion: July 25, 2024
Disclosure Updated Date: May 21, 2024

ASN
American Society of Nephrology
JASN
Journal of the American Society of Nephrology
KIDNEY360
Accessing Our World From Every Angle
CJASN
Clinical Journal of the American Society of Nephrology

ASN Journal Disclosure Form

As per ASN journal policy, I have disclosed any financial relationships or commitments I have held in the past 36 months as included below. I have listed my Current Employer below to indicate there is a relationship requiring disclosure. If no relationship exists, my Current Employer is not listed.

J. Raimann reports the following:
Employer: Renal Research Institute, a wholly owned subsidiary of Fresenius Medical Care; Ownership Interest: owning shares of stock in Fresenius Medical Care; and Other Interests or Relationships: Member of the Board of Directors "Easy Water for Everyone" (501c3).

I understand that the information above will be published within the journal article, if accepted, and that failure to comply and/or to accurately and completely report the potential financial conflicts of interest could lead to the following: 1) Prior to publication, article rejection, or 2) Post-publication, sanctions ranging from, but not limited to, issuing a correction, reporting the inaccurate information to the authors' institution, banning authors from submitting work to ASN journals for varying lengths of time, and/or retraction of the published work.

Name: Jochen G. Raimann
Manuscript ID: K360-2024-000265R1
Manuscript Title: The role of Kt/V and creatinine clearance on assisting optimization of serum phosphorus levels among patients on PD.
Date of Completion: July 25, 2024
Disclosure Updated Date: May 15, 2024

ASN American Society of Nephrology | JASN Journal of the American Society of Nephrology | KIDNEY360 Accessing Our World From Every Angle | CJASN Clinical Journal of the American Society of Nephrology

ASN Journal Disclosure Form

As per ASN journal policy, I have disclosed any financial relationships or commitments I have held in the past 36 months as included below. I have listed my Current Employer below to indicate there is a relationship requiring disclosure. If no relationship exists, my Current Employer is not listed.

S. Sharma reports the following:
Employer: Mount Sinai Hospital

I understand that the information above will be published within the journal article, if accepted, and that failure to comply and/or to accurately and completely report the potential financial conflicts of interest could lead to the following: 1) Prior to publication, article rejection, or 2) Post-publication, sanctions ranging from, but not limited to, issuing a correction, reporting the inaccurate information to the authors' institution, banning authors from submitting work to ASN journals for varying lengths of time, and/or retraction of the published work.

Name: Shuchita Sharma
Manuscript ID: K360-2024-000265R2
Manuscript Title: The role of Kt/V and creatinine clearance on assisting optimization of serum phosphorus levels among patients on PD
Date of Completion: September 17, 2024
Disclosure Updated Date: May 22, 2024

ASN American Society of Nephrology | JASN Journal of the American Society of Nephrology | KIDNEY360 Accessing Our World From Every Angle | CJASN Clinical Journal of the American Society of Nephrology

ASN Journal Disclosure Form

As per ASN journal policy, I have disclosed any financial relationships or commitments I have held in the past 36 months as included below. I have listed my Current Employer below to indicate there is a relationship requiring disclosure. If no relationship exists, my Current Employer is not listed.

J. Uribarri reports the following:
Employer: Icahn School of Medicine at Mount Sinai

I understand that the information above will be published within the journal article, if accepted, and that failure to comply and/or to accurately and completely report the potential financial conflicts of interest could lead to the following: 1) Prior to publication, article rejection, or 2) Post-publication, sanctions ranging from, but not limited to, issuing a correction, reporting the inaccurate information to the authors' institution, banning authors from submitting work to ASN journals for varying lengths of time, and/or retraction of the published work.

Name: Jaime Uribarri
Manuscript ID: K360-2024-000265R1
Manuscript Title: "The role of Kt/V and creatinine clearance on assisting optimization of serum phosphorus levels among patients on PD.
Date of Completion: July 25, 2024
Disclosure Updated Date: July 23, 2024
