# Supplementary material for: The Role of Kt/V and Creatinine Clearance on Assisting Optimization of Serum Phosphorus Levels among Patients on Peritoneal Dialysis
Source: Kidney360. 2024 Oct 11;6(1):105–11. doi: 10.34067/KID.0000000618 (PMC11793178; doi:10.34067/KID.0000000618)

Supplementary Figure 1. Distribution of serum phosphate levels across data sources.

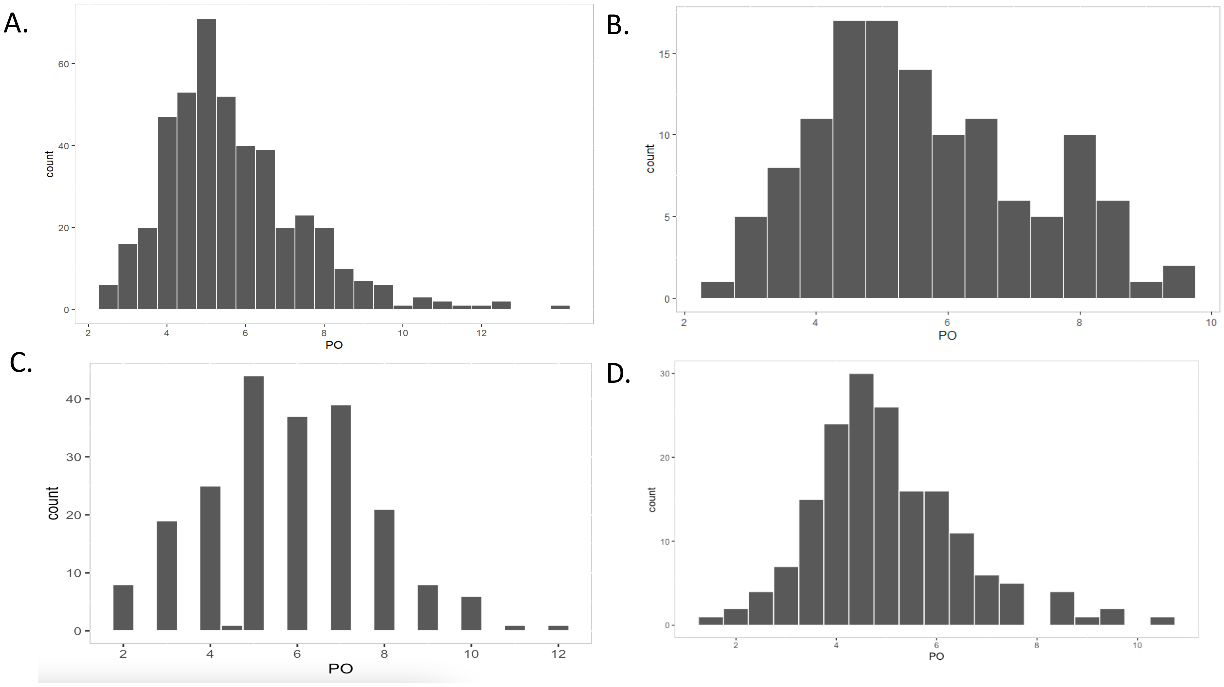

A: Renal Research Institute. B: Mount Sinai. C: Guadalajara. D: BRAZPD

Supplementary Figure 2. Distribution of Kt/V per CrCl by serum phosphate levels in the FMC-LATAM cohort.

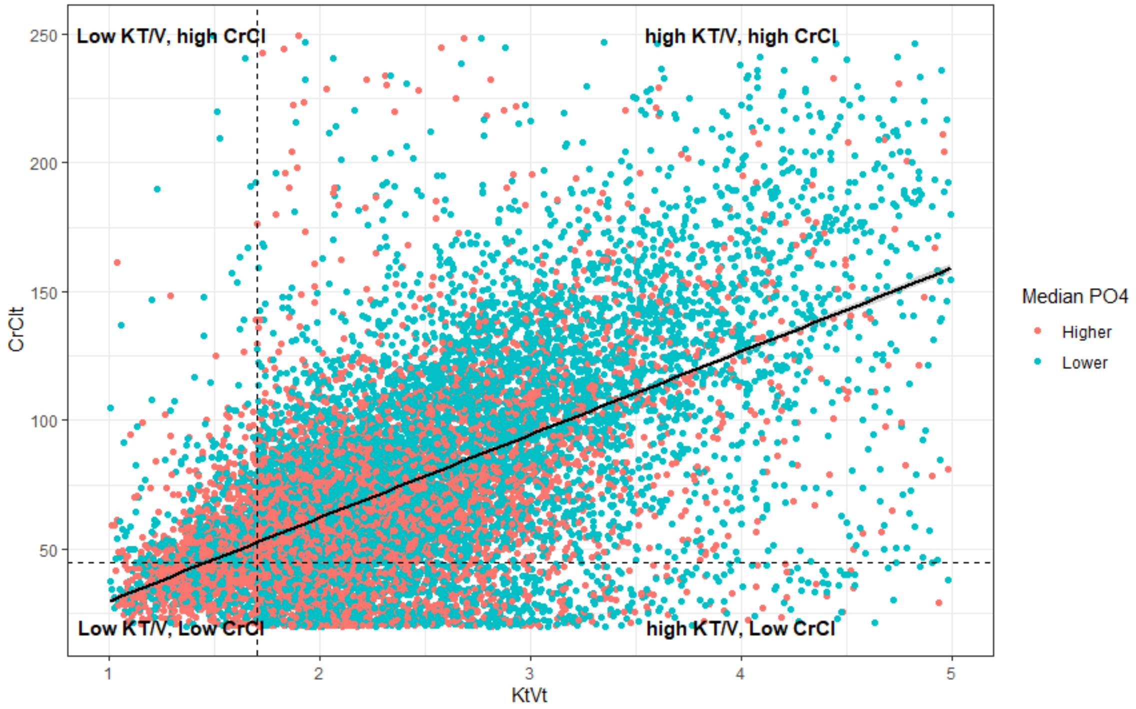

Supplementary Figure 3. Distribution of Kt/V per CrCl by serum phosphate levels in the BRAZPD cohort.

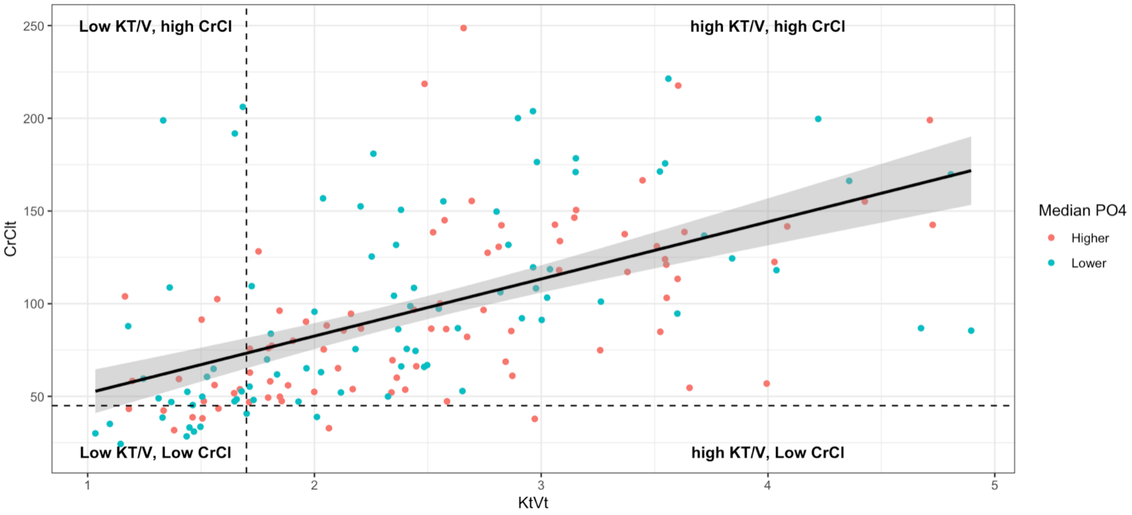

Supplementary Figure 4. Distribution of Kt/V per CrCl by serum phosphate levels in the RRI cohort.

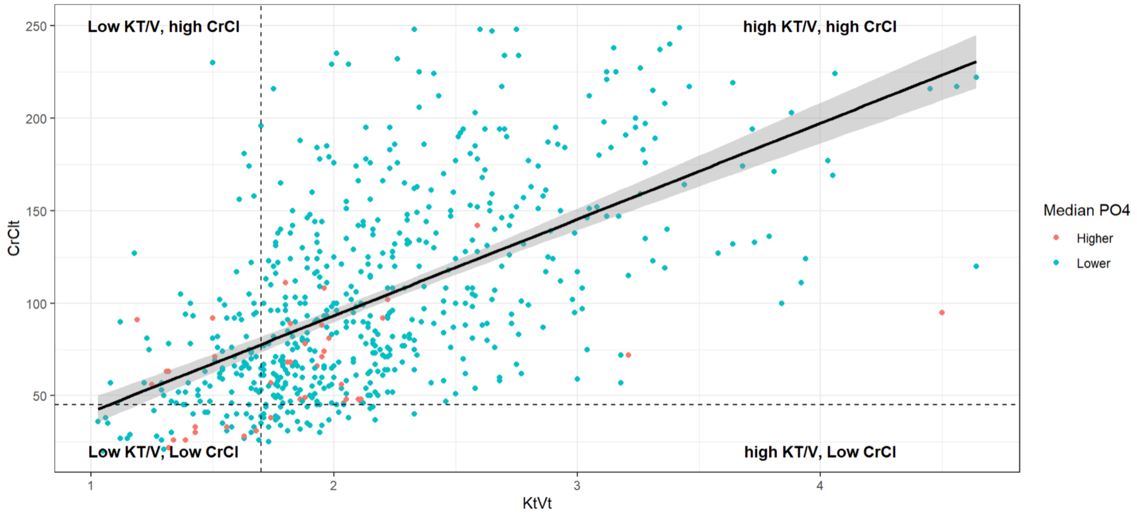

Supplementary Figure 5. Distribution of Kt/V per CrCl by serum phosphate levels in the Mount Sinai cohort.

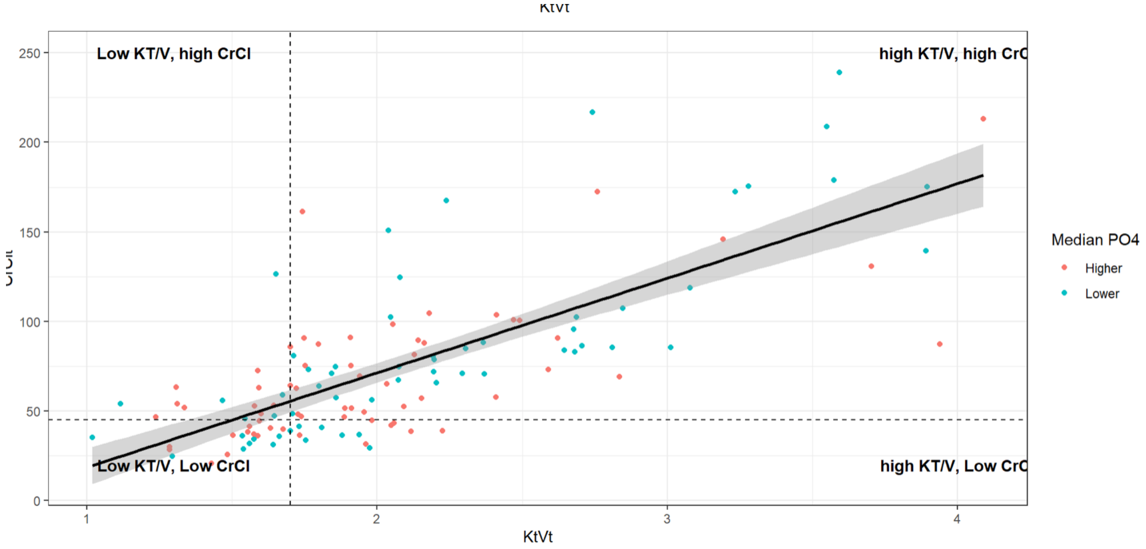

Supplementary Figure 6. Distribution of Kt/V per CrCl by serum phosphate levels in the Guadalajara cohort.

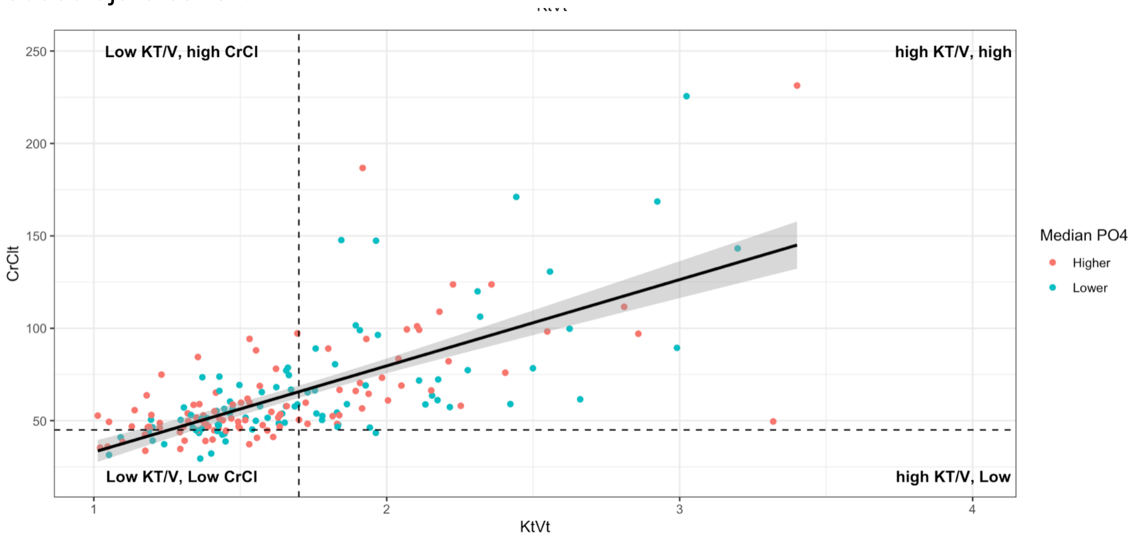

Supplement: Supplementary file 2 [file kidney360-6-105-s002.pdf]
